# Contamination and Risk Assessment of Heavy Metals in Lake Bed Sediment of a Large Lake Scenic Area in China

**DOI:** 10.3390/ijerph13070741

**Published:** 2016-07-21

**Authors:** Li Wan, Liang Xu, Yongsheng Fu

**Affiliations:** 1 Faculty of Geoscience and Environmental Engineering, Southwest Jiaotong University, No. 999 Xian Road, Chengdu 611756, China; happyviny@126.com; 2 Jilin Institute of Chemical Technology, Jilin City 132022, China; suncaiyun1985@126.com

**Keywords:** heavy metals, bed sediment, Songhua lake, ecological risk assessment, human health risk assessment

## Abstract

The exposure of heavy metals to lake bed sediment of scenic areas may pose risks on aquatic ecosystems and human health, however very few studies on risk assessment have been reported for scenic areas. Accordingly, this study determined concentration levels, and assessed contamination characteristics and risks, of heavy metals in lake bed sediment of National Scenic Areas Songhuahu (NSAS) in China. The concentrations of Zn, Cr, Pb, Ni, and Cu were determined in 29 bed sediment samples. Results showed that the mean values of Zn, Cr, Pb, Ni, and Cu were 92.69, 90.73, 38.29, 46.77, and 49.44 mg/kg, respectively. Pearson correlation coefficients indicated that organic matter was a major factor influencing distribution of heavy metals. The results for enrichment factors indicated that contamination rates and anthropogenic inputs of single heavy metals decreased in the order Cu > Ni > Pb > Cr > Zn; results of Nemerow integrated pollution index suggested that 72.41% of sampling sites were exposed to low to moderately integrated pollution, and 27.59% of sampling sites were exposed to strongly integrated pollution. According to results for potential ecological risk index, ecological risks of single and all the heavy metals in bed sediment from all the sampling sites were low. Human risks were assessed with hazardous quotients, and the results suggested that exposure of heavy metals to bed sediment posed no or little risk to human health, and the pathway of ingestion significantly contributed to human health risks.

## 1. Introduction

The contamination of heavy metals in rivers or lakes has drawn increasing attention [1], as heavy metals have characteristics of potential toxicity, persistence, and non-biodegradation which pose potential risks on aquatic ecosystems and human health [2,3,4]. In an aquatic ecosystem, sediment is an important component, acting as a major sink for heavy metals due to their great tendency of adsorbing onto solid phases [5,6]. Thus, bed sediment is the main carrier of heavy metals in lakes or rivers [7] and is regarded as an effective indicator of heavy metal pollution [8,9]. However, once toxic levels of heavy metals are reached in organisms, diversity of benthic organisms and reproduction rates can decrease and growth rates can even be reduced [10,11], causing an adverse effect on aquatic ecosystems. In addition, heavy metals may enter and accumulate in the human body through ingestions or dermal contacts, and when toxic levels of heavy metals are reached in the human body, adverse effects are posed to human health.

Scenic areas are places that a large number of people visit, especially on national holidays. The exposure of pollutants in environmental media may have adverse effects to public health, but very few studies on contamination risk assessment of pollutants have been reported for the scenic areas. In this study, we selected National Scenic Area Songhuahu (NSAS) as the study area, as it is one of the most famous lake scenic areas in China. NSAS has a lake scenic area of 554 square kilometers and is located in Jilin City of China where the region is subjected to a semi-humid continental monsoon climate. The lake scenic area is long and narrow, and the maximum water-storage is 10.8 billion cubic meters. NSAS is fed by Songhua River. However, with rapid development of industry and urbanization, Songhua River has been receiving an increasing amount of industrial and domestic effluents, vehicular emissions, and wastewater produced by mining and refining activities [12,13,14], which may pose potential contamination risks of heavy metals on aquatic ecosystem of NSAS [15]. NSAS is famous for its lake scenery, and a large number of people visit every year. However, potential contamination of heavy metals in the aquatic environment may pose risks to human health and the aquatic ecosystem.

Contamination risk assessment of pollutants is fundamental for contamination control and management, so routinely monitoring concentrations of pollutants and evaluating contamination risks is essential [16]. Accordingly, the main aims of this work are to determine concentration levels of heavy metals in lake bed sediment of NSAS, evaluate contamination characteristics, and assess risks of heavy metals to the aquatic ecosystem and human health. This study will provide valuable information on contamination control and management.

## 2. Materials and Methods

NSAS—with a lake scenic area of 554 square kilometers—is located in Jilin City, Jilin Province of China, where the region is subjected to a semi-humid continental monsoon climate. The lake scenic area is long and narrow, and the maximum water-storage is 10.8 billion cubic meters. NSAS is fed by Songhua River where potential sources of heavy metals are located.

**Figure 1:**
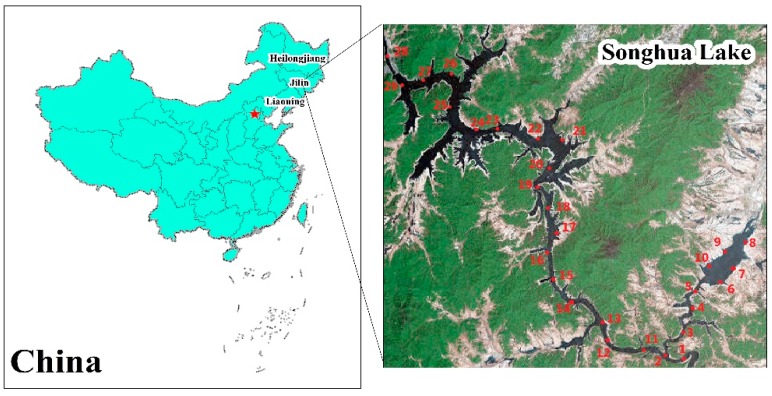
The locations of National Scenic Areas Songhuahu (NSAS) and sampling sites.

On 1–3 May 2016, a total of 29 bed sediment samples (5–10 cm) were collected to evaluate comprehensive pollution of heavy metals in the aquatic ecosystem; the sampling sites are represented in Figure 1. All the sampling sites are located along Songhua lake where a large number of tourists visit and sanatoriums and hotels are located. Sites 1 and 2 are located in the inflow-river area of Songhua lake, and site 29 is located in the outflow-river area of Songhua lake. Sites 3–10 are located in places where there are less anthropogenic activities, and sites 11–28 are located in developed-tourism areas. To reduce randomicity, each bed sediment sample was thoroughly mixed with three subsamples, and the distance of every two subsamples was at least one hundred meters away. Bed sediment samples were collected with an Ekman grab sampler, placed in polyethylene plastic bags, labeled, immediately transported to the lab, and kept under 4 °C until analyses were performed.

### 2.1. Sediment Analyses

Each bed sediment sample was air dried, ground with a mortar, and passed through a 100-mesh sieve. Then, 1 g of pretreated sediment sample was digested with HClO_4_-HNO_3_-HF [17,18], and the concentrations of Zn, Cr, Pb, Ni, and Cu in the extracts were determined using an atom absorption spectrophotometer (Z-5000, HITACHI, Tokyo, Japan).

The values of sediment pH were measured (sediment:water 1:2.5 dry weight/volume) using a pH-meter (pHS-3B, Leici, Shanghai, China). Organic matter contents were determined by the Walkey-Black method [19].

### 2.2. Quality Insurance

Before pretreatment of bed sediment samples, the recovery rates for heavy metals were conducted. The results showed that recovery rates were 93%–99% for Zn, 92%–110% for Cr, 90%–105% for Pb, 88%–97% for Ni, and 93%–109% for Cu. All the chemical analyses and control experiments were conducted in duplicate, and the standard deviations were all within ±5%.

### 2.3. Data Analysis

#### 2.3.1. Enrichment Factors

This study applied enrichment factors (EFs) to characterize the contamination rates and assess anthropogenic inputs of single heavy metals [20]. The values of EFs were calculated with Equation (1):

EFs = (C_n_/B_n_ − 1) × 100 (1)

where C_n_ is the monitoring concentration of specific heavy metal in bed sediment; B_n_ is background concentration of specific heavy metal in bed sediment. The background concentrations were obtained from a published article [21]. The values of EFs ≤ 0 indicate no anthropogenic inputs of heavy metals in bed sediment samples, and the values of EFs > 0 indicate anthropogenic inputs of heavy metals in bed sediment samples.

#### 2.3.2. Nemerow Integrated Pollution Index

To evaluate integrated contamination characteristics of all the heavy metals in bed sediment, Nemerow integrated pollution (P_n_) index was applied. P_n_ was expressed as Equation (3), and the P_n_ levels are illustrated as Table 1 [22].

P_i_ = C_i_/B_i_ (2)

P_n_ = {[(P_iav_)^2^ + (P_imax_)^2^]/2}^1/2^ (3)

where C_i_ is the monitoring concentration of specific heavy metal; B_i_ is the background concentration of specific value; P_iav_ is the average value of single pollution indexes for all the heavy metals; P_imax_ is the maximum value of the single pollution indexes for all the heavy metals [23].

#### 2.3.3. Potential Ecological Risk Index

In this study, the potential ecological risk index (PERI) was applied to evaluate potential ecological risks (ER) of single heavy metals and all the heavy metals [24]. The values of ER for single heavy metals and values of PERI for all the heavy metals were respectively calculated as Equations (4) and (5):

ER = Ti × C_i0_/B_in_ (4)

PERI = ∑ Ti × C_i0_/B_i_ (5)

where C_i0_ is the monitoring concentration of specific heavy metal in bed sediment; B_in_ is the background concentration of specific heavy metal in bed sediment; and T_i_ is the toxic-response factor for specific heavy metal, where the values for Zn, Cr, Pb, Ni, and Cu are 1, 2, 5, 5, and 5, respectively. The potential ecological risk levels for single heavy metals are regarded as follows: low risk (ER < 40), moderate risk (40 ≤ ER < 80), considerable risk (80 ≤ ER < 160), high risk (160 ≤ ER < 320), and very high risk (ER ≥ 320). The potential comprehensive ecological risk levels for all the heavy metals are regarded as follows: low ecological risk (PERI < 150), moderate ecological risk (150 ≤ PERI < 300), considerable ecological risk (300 ≤ PERI < 600), and very high ecological risk (PERI ≥ 600) [6].

#### 2.3.4. Human Health Risk Assessment

In general, the major pathways of ingestion and dermal contact are considered in human health risk assessment [25]. The exposures through ingestion and dermal contact were respectively calculated using Equations (6) and (7):

Exp_ing_ = (C_i_ × IR × CF × EF × ED)/(BW × AT) (6)

Exp_der_ = (C_i_ × CF × SA × AF × ABS × EF × ED)/(BW × AT) (7)

where Exp_ing_ is the exposure of ingestion from sediment; C_i_ is the monitoring concentration of a specific heavy metal in the bed sediment; IR is the ingestion rate (114 mg/day); CF is the unit conversion factor (10^−6^ kg/mg); EF is the exposure frequency (350 days/year); ED is the exposure duration (30 years); BW is the body weight (70 kg); AT is the average day (10,950 days); Exp_der_ is the exposure of dermal contacts from sediment; SA is the exposed skin surface area (5700 cm^2^); AF is the adherence factor from sediment to skin (0.07 mg/cm^2^); and ABS is the dermal absorption from sediment (0.001).

Hazardous quotients (HQs) were applied to assess noncarcinogenic risks of heavy metals in bed sediment [26]. The values of HQs for pathways of ingestion and dermal contact were respectively calculated according to Equations (8) and (9):

HQ_ing/der_ = Exp_ing/der_/RfD (8)

HI = ∑ HQ_ing/der_ (9)

where RfD is the reference value for causing adverse effects on human health by specific heavy metal. The reference values for dermal contact are considered to be identical to the reference values for ingestion of heavy metals in sediment [27]. HQ_ing/der_ < 1 indicates that exposure of heavy metals to sediment may pose no or little risks on human health; HQ_ing/der_ > 1 indicates that exposure of heavy metals to sediment may pose risks on human health [28].

## 3. Results

### 3.1. Occurrence of Heavy Metals in Bed Sediment

Table 2 represents descriptive statistics of heavy metal concentrations in bed sediment of NSAS; the concentrations of Zn, Cr, Pb, Ni, and Cu were analyzed. The results showed that all five heavy metals were detected in all the samples, and the mean values of Zn, Cr, Pb, Ni, and Cu were 92.69, 90.73, 38.29, 46.77, and 49.44 mg/kg, respectively. The mean concentrations of these five heavy metals all exceeded the corresponding background concentrations. The values of coefficients of variation for five heavy metals varied from 28.85% for Zn to 54.21% for Cu.

Physicochemical parameters, sediment pH, and organic matter (OM) contents were measured. The values of sediment pH ranged from 5.46 to 8.91 with a mean value of 7.39. Most sediment samples were neutral, with 13 sediment samples of the soils having pH values between 7 and 8, eight sediment samples having pH values below 7, and eight sediment samples having pH values above 8. Organic matter contents ranged from 2.35% to 5.7% with a mean value of 3.97%. The correlations between heavy metals and sediment pH and physicochemical parameters were evaluated with Pearson correlation coefficients. As represented in Table 3, the correlations between heavy metals and pH were weak; the correlations between most of the heavy metals and OM were strongly positive.

### 3.2. Contamination Characteristics and Risk Assessment

#### 3.2.1. Enrichment Factors

EFs were applied to characterize contamination rates and assess anthropogenic inputs of single heavy metals. As represented in Table 4, most values of EFs for five heavy metals in different sampling sites were greater than zero. The values of EFs for Zn ranged from −6.63% to 188.08% with a mean value of 55.87%; the values of EFs for Cr ranged from −31.99% to 272.77% with a mean value of 80.85%; the values of EFs for Pb ranged from −22.02% to 255.94% with a mean value of 87.09%; the values of EFs for Ni ranged from −41.83% to 329.35% with a mean value of 102.72%; and the values of EFs for Cu ranged from −12.39% to 617.65% with a mean value of 175.19%.

#### 3.2.2. Nemerow Integrated Pollution Index

**Figure 2:**
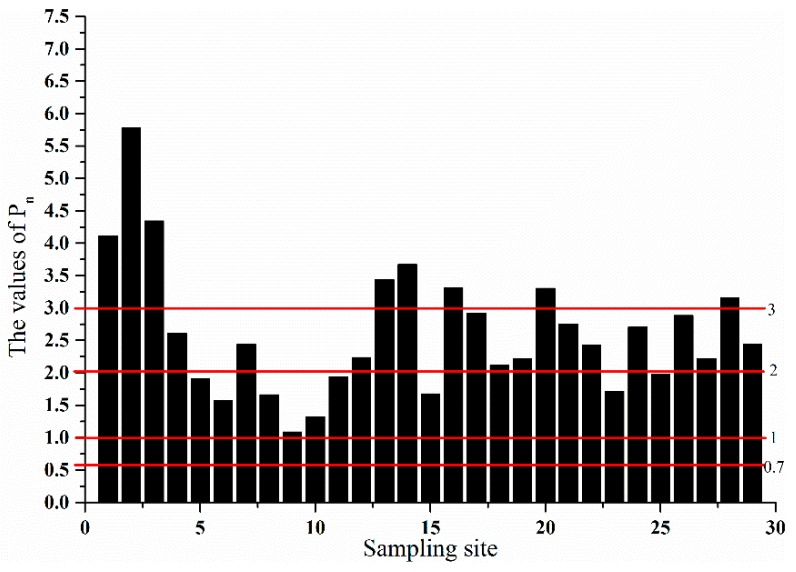
The Nemerow integrated pollution (P_n_) values of five heavy metals in different sampling sites.

To evaluate integrated pollution levels of all the heavy metals, P_n_ index was employed. Figure 2 indicates integrated pollution levels of all the heavy metals in different sampling sites. The values of P_n_ ranged from 1.09 to 5.77. The values of P_n_ in nine sampling sites were between 1 and 2; the values of P_n_ in twelve sampling sites ranged from 2 to 3; the values of P_n_ in eight sampling sites were greater than 3. The highest values of P_n_ occurred in sites 1 and 2, while the lowest values occurred in sites 9 and 10.

#### 3.2.3. Ecological Risk Assessment

**Figure 3:**
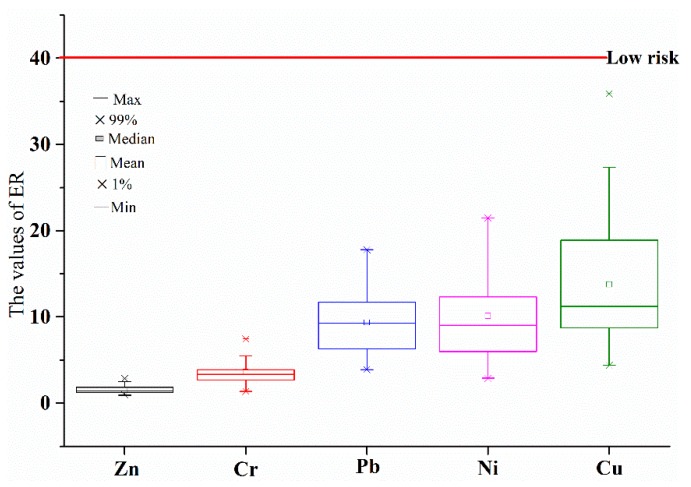
The ecological risks (ER) of single heavy metals in different sampling sites.

In this work, PERI was used to assess ecological risks of all the heavy metals and single heavy metals in different sampling sites. As represented in Figure 3, the values of ER for five heavy metals in all the sampling sites were smaller than 40.

**Figure 4:**
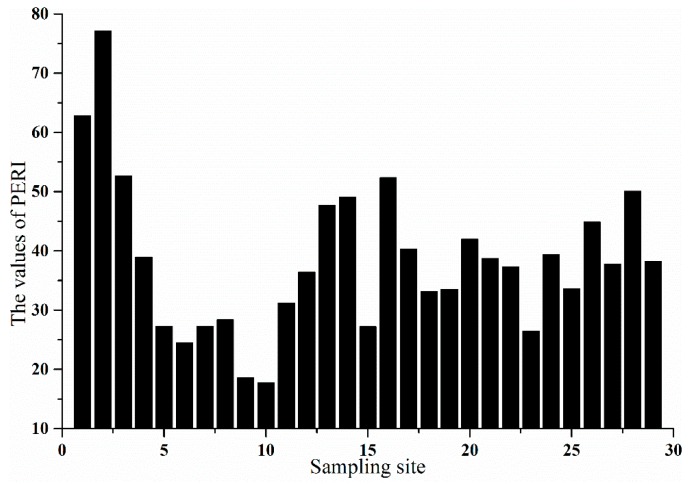
The values of potential ecological risk index (PERI) in different sampling sites.

Figure 4 indicates comprehensive ecological risks of all the heavy metals in different sampling sites. The values of PERI in all the sampling sites were smaller than 150. Among 29 sampling sites, the comprehensive ecological risks of all the heavy metals were the lowest in sites 9 and 10, whereas the highest comprehensive ecological risks existed in sites 1 and 2. The comprehensive ecological risk in site 28 was relatively higher, but the value of PERI in site 28 was below 150.

#### 3.2.4. Human Health Risk Assessment

**Figure 5:**
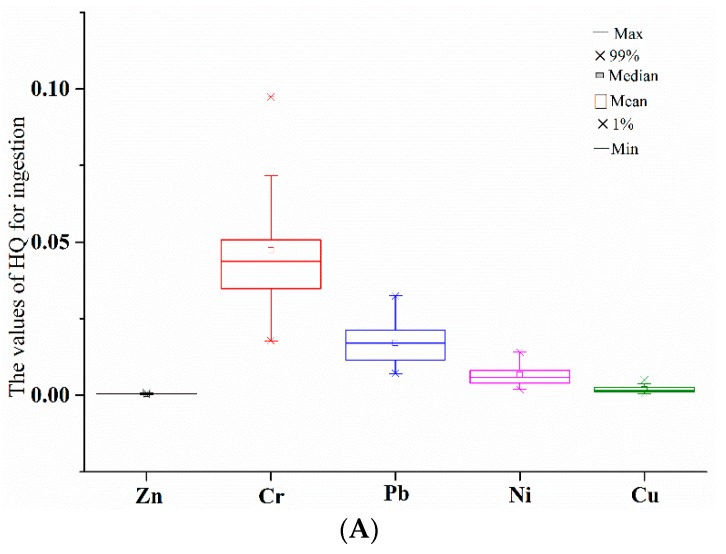

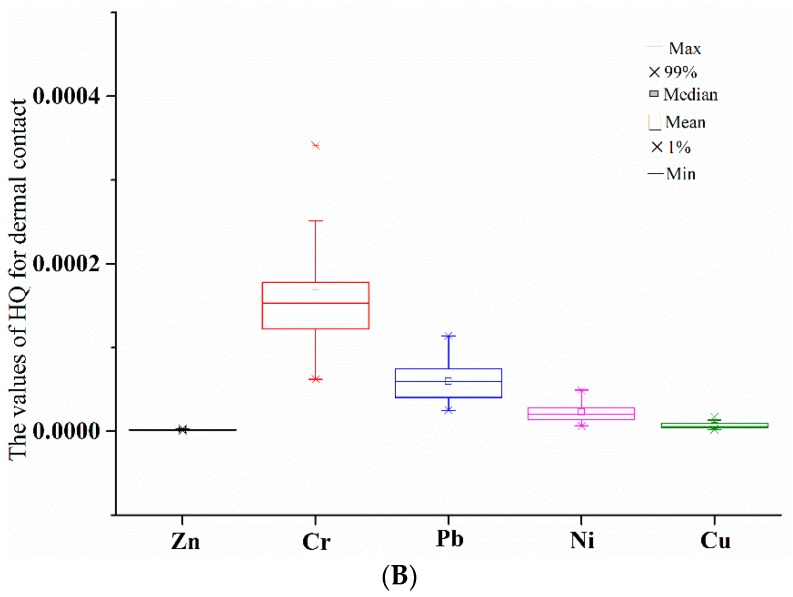
Human health risks of single heavy metals posed through pathways of ingestion (**A**) and dermal contact (**B**). HQ, hazardous quotient.

Human health risk assessment was conducted with hazardous quotients (HQs) and the results are represented in Table 5. Hazardous index (HI) is the sum of HQ. The values of HI in all the sampling sites were smaller than 1. The values of HQs for exposure of each heavy metal through pathways of ingestion and dermal contact to bed sediment from all the sampling sites were smaller than 1. As represented in Figure 5A,B, among five heavy metals, the values of HQs for ingestion and dermal contact both decreased in the order Cr > Pb > Ni > Cu > Zn. The values of HQs for exposures of all the heavy metals through ingestion were two or three orders of magnitude higher than through dermal contact.

## 4. Discussion

### 4.1. Heavy Metals in Bed Sediment of NSAS

The concentrations of heavy metals in bed sediment decreased in the order Zn > Cr > Cu > Ni > Pb. The results for CVs indicated that anthropogenic input was involved in enrichment of heavy metals in bed sediment, and decreased in the order Cu > Ni > Cr > Pb > Zn.

Most of the sediment samples were neutral pH. The pH was not a factor influencing distribution of heavy metals in bed sediment, while OM could influence distribution of heavy metals in bed sediment.

### 4.2. Contamination Characteristics and Risk Assessment

#### 4.2.1. Contamination Characteristics

The results for EFs indicated that anthropogenic inputs contributed to enrichment of heavy metals in bed sediment. The contamination rates and anthropogenic inputs of single heavy metals decreased in the order Cu > Ni > Pb > Cr > Zn, the highest rate and input of Cu were three times higher than the lowest rate and input of Zn.

According to the results of P_n_ index, the integrated pollution levels were greatly varied in different sampling sites, which was due to great variation in spatial distribution of heavy metals. Nine out of 29 sampling sites were exposed to low integrated pollution of all the heavy metals; 12 sampling sites were exposed to moderately integrated pollution; eight sampling sites had strongly integrated pollution. Generally, 72.41% of sampling sites were exposed to low to moderately integrated pollution, and 27.59% of sampling sites were exposed to strongly integrated pollution. Spatially, the pollution sources from Songhua River posed a potential pollution risk to NSAS, and the lowest integrated pollution levels occurred in the largest branch of NSAS where tourism had not been developed.

#### 4.2.2. Risk Assessment

The results for ER indicated that ecological risks of single heavy metals in all the sampling sites were low, and ecological risk levels of five heavy metals decreased in the order Cu > Ni > Pb > Cr > Zn.

The results for PERI suggested that all the sampling sites were exposed to low comprehensive ecological risks of all the heavy metals. Among 29 sampling sites, the comprehensive ecological risks of all the heavy metals were the lowest in the largest branch of NSAS, which was an undeveloped-tourism area. The highest comprehensive ecological risks existed in inflow-river area, which might be attributed to high anthropogenic inputs of heavy metals into Songhua River. The comprehensive ecological risk downstream of Songhua River was low. This could lead to the conclusion that tourism in NSAS did not pose ecological risks with heavy metals downstream of Songhua River.

Results for HQs and HIs indicated that the exposure of all the heavy metals to bed sediment through ingestion and dermal contact posed no or little risks to human health. All the sampling sites exposed no or little human health risks of each heavy metals in bed sediment. Among five heavy metals, human health risks through the pathways of ingestion and dermal contact both decreased in the order Cr > Pb > Ni > Cu > Zn, with exposures of Cr and Pb to bed sediment posed greater risks to human health. The pathway of ingestion significantly contributed to human health risks of heavy metals in bed sediment.

## 5. Conclusions

The results obtained in this work offer information on concentration levels, contamination characteristics, and ecological and human health risks of heavy metals in bed sediment of NSAS. The concentrations of Zn, Cr, Pb, Ni and Cu were determined in a total of 29 bed sediment samples. The results show that mean values of Zn, Cr, Pb, Ni, and Cu were 92.69, 90.73, 38.29, 46.77 and 49.44 mg/kg, respectively. According to the results of EFs, contamination rates and anthropogenic inputs of single heavy metals decreased in the order Cu > Ni > Pb > Cr > Zn; the results of P_n_ index indicated that integrated pollution of all the heavy metals in 72.41% of sampling sites were low to moderate, and integrated pollution in 27.59% of sampling sites were strong. Results for PERI indicated that ecological risks of single and all the heavy metals in bed sediment from all the sampling sites were low. The results of HQs and HIs suggested exposures of heavy metals to bed sediment posed no or little risks to human health, and the pathway of ingestion significantly contributed to human health risks.

## Figures and Tables

**Table 1:** Illustration Nemerow integrated pollution (P_n_) levels related to sediment quality.

Values of P_n_	Level of P_n_	Sediment Quality
0–0.7	1	Unpolluted
0.7–1	2	Warning limit
1–2	3	Low polluted
2–3	4	Moderately polluted
>3	5	Strongly polluted

**Table 2:** Descriptive statistics of heavy metal concentrations in bed sediment of National Scenic Area Songhuahu (NSAS).

	Zn (mg/kg)	Cr (mg/kg)	Pb (mg/kg)	Ni (mg/kg)	Cu (mg/kg)	pH	^c^ OM (%)
min	55.53	34.12	15.96	13.42	15.74	5.46	2.35
max	171.32	187.02	72.85	99.05	128.94	8.91	5.7
mean	92.69	90.73	38.29	46.77	49.44	7.39	3.97
^a^ CV (%)	28.85	39.71	38.22	48.38	54.21	12.99	21.46
^b^ BC	59.47	50.17	20.46	23.07	17.96		

^a^ Coefficients of variation; ^b^ Background concentrations; ^c^ Organic matter.

**Table 3:** Pearson correlations between heavy metals and physicochemical parameters.

	pH	OM
Zn	−0.186	0.387 *
Cr	0.038	0.286
Pb	0.119	0.343
Ni	−0.275	0.374 *
Cu	0.071	0.454 *

* Denotes that correlation is significant at the 0.05 level (2-tailed).

**Table 4:** The values of enrichment factors (EFs; %) for five heavy metals in bed sediment of NSAS.

Sampling Site	Zn	Cr	Pb	Ni	Cu
1	150.93	272.77	107.46	274.64	374.98
2	188.08	194.04	119.62	329.35	617.65
3	104.04	201.97	7.25	137.75	446.61
4	30.35	73.89	152.70	19.77	211.29
5	83.15	113.73	9.98	−0.95	113.84
6	9.87	33.43	36.85	80.19	−3.04
7	26.84	68.01	−22.02	−35.85	210.68
8	−6.63	81.06	33.97	67.45	75.21
9	5.43	12.60	12.08	6.07	−12.39
10	19.74	−31.99	−12.93	−41.83	58.57
11	59.64	58.28	26.25	115.56	86.62
12	75.26	142.73	104.82	59.60	131.81
13	26.69	215.77	176.98	7.02	317.10
14	50.36	67.31	152.75	71.30	359.95
15	13.01	84.19	16.33	50.93	80.78
16	47.18	30.40	85.42	284.14	295.78
17	89.04	54.02	255.94	85.26	65.25
18	29.34	39.98	103.45	146.34	30.96
19	48.24	2.91	133.74	2.43	163.04
20	3.28	48.02	119.13	19.20	321.22
21	77.27	13.39	33.97	235.59	124.30
22	100.99	53.94	85.91	178.67	79.11
23	43.33	89.75	7.20	67.88	48.83
24	29.65	94.40	55.13	104.55	224.87
25	40.42	53.82	83.32	80.75	117.51
26	108.95	74.05	229.46	234.50	22.11
27	86.33	5.06	143.42	86.17	146.56
28	36.59	22.24	152.99	194.06	277.86
29	42.85	174.95	114.54	118.34	93.41
Max	188.08	272.77	255.94	329.35	617.65
Min	−6.63	−31.99	−22.02	−41.83	−12.39
Mean	55.87	80.85	87.09	102.72	175.19

**Table 5:** Human health risks of five heavy metals in bed sediment of National Scenic Area Songhuahu (NSAS).

Sampling	Ingestion					Dermal					HI
Site	Zn	Cr	Pb	Ni	Cu	Zn	Cr	Pb	Ni	Cu	
1	7.77 × 10^−4^	9.74 × 10^−2^	1.89 × 10^−2^	1.23 × 10^−2^	3.33 × 10^−3^	2.72 × 10^−6^	3.41 × 10^−4^	6.63 × 10^−5^	4.29 × 10^−5^	1.17 × 10^−5^	1.33 × 10^−1^
2	8.92 × 10^−4^	7.68 × 10^−2^	2.01 × 10^−2^	1.41 × 10^−2^	5.03 × 10^−3^	3.12 × 10^−6^	2.69 × 10^−4^	7.02 × 10^−5^	4.92 × 10^−5^	1.76 × 10^−5^	1.17 × 10^−1^
3	6.32 × 10^−4^	7.89 × 10^−2^	9.79 × 10^−3^	7.79 × 10^−3^	3.83 × 10^−3^	2.21 × 10^−6^	2.76 × 10^−4^	3.43 × 10^−5^	2.73 × 10^−5^	1.34 × 10^−5^	1.01 × 10^−1^
4	4.04 × 10^−4^	4.54 × 10^−2^	2.31 × 10^−2^	3.92 × 10^−3^	2.18 × 10^−3^	1.41 × 10^−6^	1.59 × 10^−4^	8.08 × 10^−5^	1.37 × 10^−5^	7.64 × 10^−6^	7.53 × 10^−2^
5	5.67 × 10^−4^	5.58 × 10^−2^	1.00 × 10^−2^	3.24 × 10^−3^	1.50 × 10^−3^	1.98 × 10^−6^	1.95 × 10^−4^	3.52 × 10^−5^	1.14 × 10^−5^	5.25 × 10^−6^	7.14 × 10^−2^
6	3.40 × 10^−4^	3.48 × 10^−2^	1.25 × 10^−2^	5.90 × 10^−3^	6.80 × 10^−4^	1.19 × 10^−6^	1.22 × 10^−4^	4.37 × 10^−5^	2.07 × 10^−5^	2.38 × 10^−6^	5.45 × 10^−2^
7	3.93 × 10^−4^	4.39 × 10^−2^	7.12 × 10^−3^	2.10 × 10^−3^	2.18 × 10^−3^	1.37 × 10^−6^	1.54 × 10^−4^	2.49 × 10^−5^	7.35 × 10^−6^	7.63 × 10^−6^	5.59 × 10^−2^
8	2.89 × 10^−4^	4.7 × 10^−2^	1.22 × 10^−2^	5.4 × 10^−3^	1.23 × 10^−3^	1.01 × 10^−6^	1.66 × 10^−4^	4.28 × 10^−5^	1.92 × 10^−5^	4.30 × 10^−6^	6.68 × 10^−2^
9	3.26 × 10^−4^	2.94 × 10^−2^	1.02 × 10^−2^	3.47 × 10^−3^	6.15 × 10^−4^	1.14 × 10^−6^	1.03 × 10^−4^	3.58 × 10^−5^	1.22 × 10^−5^	2.15 × 10^−6^	4.42 × 10^−2^
10	3.71 × 10^−4^	1.78 × 10^−2^	7.95 × 10^−3^	1.91 × 10^−3^	1.11 × 10^−3^	1.30 × 10^−6^	6.22 × 10^−5^	2.78 × 10^−5^	6.67 × 10^−5^	3.89 × 10^−6^	2.92 × 10^−2^
11	4.94 × 10^−4^	4.13 × 10^−2^	1.15 × 10^−2^	7.06 × 10^−3^	1.31 × 10^−3^	1.73 × 10^−6^	1.45 × 10^−4^	4.04 × 10^−5^	2.47 × 10^−5^	4.58 × 10^−6^	6.19 × 10^−2^
12	5.43 × 10^−4^	6.34 × 10^−2^	1.87 × 10^−2^	5.23 × 10^−3^	1.63 × 10^−3^	1.90 × 10^−6^	2.22 × 10^−4^	6.55 × 10^−5^	1.83 × 10^−5^	5.69 × 10^−6^	8.98 × 10^−2^
13	3.92 × 10^−4^	8.25 × 10^−2^	2.53 × 10^−2^	3.51 × 10^−3^	2.93 × 10^−3^	1.37 × 10^−6^	2.89 × 10^−4^	8.85 × 10^−5^	1.23 × 10^−5^	1.02 × 10^−6^	1.15 × 10^−1^
14	4.65 × 10^−4^	4.37 × 10^−2^	2.31 × 10^−2^	5.61 × 10^−3^	3.23 × 10^−3^	1.63 × 10^−6^	1.53 × 10^−4^	8.08 × 10^−5^	1.96 × 10^−5^	1.13 × 10^−6^	7.63 × 10^−2^
15	3.50 × 10^−4^	4.81 × 10^−2^	1.06 × 10^−2^	4.94 × 10^−3^	1.27 × 10^−3^	1.22 × 10^−6^	1.68 × 10^−4^	3.72 × 10^−5^	1.73 × 10^−5^	4.44 × 10^−6^	6.55 × 10^−2^
16	4.56 × 10^−4^	3.41 × 10^−2^	1.69 × 10^−2^	1.26 × 10^−2^	2.78 × 10^−3^	1.59 × 10^−6^	1.19 × 10^−4^	5.93 × 10^−5^	4.40 × 10^−5^	9.72 × 10^−6^	6.70 × 10^−2^
17	5.85 × 10^−4^	4.02 × 10^−2^	3.25 × 10^−2^	6.07 × 10^−3^	1.16 × 10^−3^	2.05 × 10^−6^	1.41 × 10^−4^	1.14 × 10^−5^	2.12 × 10^−5^	4.06 × 10^−6^	8.08 × 10^−2^
18	4.00 × 10^−4^	3.66 × 10^−2^	1.86 × 10^−2^	8.07 × 10^−3^	9.19 × 10^−4^	1.40 × 10^−6^	1.28 × 10^−4^	6.50 × 10^−5^	2.82 × 10^−5^	3.22 × 10^−6^	6.48 × 10^−2^
19	4.59 × 10^−4^	2.69 × 10^−2^	2.13 × 10^−2^	3.35 × 10^−3^	1.85 × 10^−3^	1.61 × 10^−6^	9.41 × 10^−4^	7.47 × 10^−5^	1.17 × 10^−5^	6.46 × 10^−6^	5.41 × 10^−2^
20	3.20 × 10^−4^	3.87 × 10^−2^	2.00 × 10^−2^	3.90 × 10^−3^	2.95 × 10^−3^	1.12 × 10^−6^	1.35 × 10^−4^	7.00 × 10^−5^	1.37 × 10^−5^	1.03 × 10^−6^	6.61 × 10^−2^
21	5.49 × 10^−4^	2.96 × 10^−2^	1.22 × 10^−2^	1.10 × 10^−2^	1.57 × 10^−3^	1.92 × 10^−6^	1.04 × 10^−4^	4.28 × 10^−5^	3.85 × 10^−5^	5.51 × 10^−6^	5.52 × 10^−2^
22	6.22 × 10^−4^	4.02 × 10^−2^	1.70 × 10^−2^	9.13 × 10^−3^	1.26 × 10^−3^	2.18 × 10^−6^	1.41 × 10^−4^	5.94 × 10^−5^	3.19 × 10^−5^	4.40 × 10^−6^	6.84 × 10^−2^
23	4.44 × 10^−4^	4.96 × 10^−2^	9.79 × 10^−3^	5.50 × 10^−3^	1.04 × 10^−3^	1.55 × 10^−6^	1.73 × 10^−4^	3.43 × 10^−5^	1.92 × 10^−5^	3.65 × 10^−6^	6.66 × 10^−2^
24	4.01 × 10^−4^	5.08 × 10^−2^	1.42 × 10^−2^	6.70 × 10^−3^	2.28 × 10^−3^	1.40 × 10^−6^	1.78 × 10^−4^	4.96 × 10^−5^	2.34 × 10^−5^	7.98 × 10^−6^	7.46 × 10^−2^
25	4.35 × 10^−4^	4.02 × 10^−2^	1.67 × 10^−2^	5.92 × 10^−3^	1.53 × 10^−3^	1.52 × 10^−6^	1.41 × 10^−4^	5.86 × 10^−5^	2.07 × 10^−5^	5.34 × 10^−6^	6.50 × 10^−2^
26	6.47 × 10^−4^	4.55 × 10^−2^	3.01 × 10^−2^	1.10 × 10^−2^	8.57 × 10^−4^	2.26 × 10^−6^	1.59 × 10^−4^	1.05 × 10^−4^	3.83 × 10^−5^	3.00 × 10^−6^	8.83 × 10^−2^
27	5.77 × 10^−4^	2.74 × 10^−2^	2.22 × 10^−2^	6.10 × 10^−3^	1.73 × 10^−3^	2.02 × 10^−6^	9.60 × 10^−4^	7.78 × 10^−5^	2.13 × 10^−5^	6.05 × 10^−6^	5.83 × 10^−2^
28	4.23 × 10^−4^	3.19 × 10^−2^	2.31 × 10^−2^	9.63 × 10^−3^	2.65 × 10^−3^	1.48 × 10^−6^	1.12 × 10^−4^	8.09 × 10^−5^	3.37 × 10^−5^	9.28 × 10^−6^	6.80 × 10^−2^
29	4.42 × 10^−4^	7.18 × 10^−2^	1.96 × 10^−2^	7.15 × 10^−3^	1.36 × 10^−3^	1.55 × 10^−6^	2.51 × 10^−4^	6.86 × 10^−5^	2.50 × 10^−5^	4.75 × 10^−6^	1.01 × 10^−2^

HI, hazardous index.
